# A magnetically controlled chemical–mechanical polishing (MC-CMP) approach for fabricating channel-cut silicon crystal optics for the High Energy Photon Source

**DOI:** 10.1107/S1600577522011122

**Published:** 2023-01-01

**Authors:** Zhen Hong, Qianshun Diao, Wei Xu, Qingxi Yuan, Junliang Yang, Zhongliang Li, Yongcheng Jiang, Changrui Zhang, Dongni Zhang, Fang Liu, Xiaowei Zhang, Peng Liu, Ye Tao, Weifan Sheng, Ming Li, Yidong Zhao

**Affiliations:** a Beijing Synchrotron Radiation Facility, Institute of High Energy Physics, Chinese Academy of Sciences, Beijing 100049, People’s Republic of China; b University of Chinese Academy of Sciences, Chinese Academy of Sciences, Beijing 100049, People’s Republic of China; c Shanghai Synchrotron Radiation Facility, Shanghai Advanced Research Institute, Chinese Academy of Sciences, Shanghai 201800, People’s Republic of China; RIKEN SPring-8 Center, Japan

**Keywords:** channel-cut crystal, MC-CMP, high-accuracy roughness, residue-stress free, scratch- and speckle-free

## Abstract

Crystal monochromators are indispensable optical components for the majority of beamlines at synchrotron radiation facilities. Channel-cut monochromators are sometimes chosen to filter monochromatic X-ray beams by virtue of their ultrahigh angular stability. Nevertheless, high-accuracy polishing on the inner diffracting surfaces remains challenging, thus hampering their performance in preserving the coherence or wavefront of the photon beam. Herein, a magnetically controlled chemical–mechanical polishing (MC-CMP) approach has been successfully developed for fine polishing of the inner surfaces of channel-cut crystals. This MC-CMP process relieves the constraints of narrow working space dictated by small offset requirements and achieves near-perfect polishing on the surface of the crystals. Using this method, a high-quality surface with roughness of 0.614 nm (root mean square, r.m.s.) is obtained in a channel-cut crystal with 7 mm gap designed for beamlines at the High Energy Photon Source, a fourth-generation synchrotron radiation source under construction. On-line X-ray topography and rocking-curve measurements indicate that the stress residual layer on the crystal surface was removed. Firstly, the measured rocking-curve width is in good agreement with the theoretical value. Secondly, the peak reflectivity is very close to theoretical values. Thirdly, topographic images of the optics after polishing were uniform without any speckle or scratches. Only a nearly 2.5 nm-thick SiO_2_ layer was observed on the perfect crystalline matrix from high-resolution transmission electron microscopy photographs, indicating that the structure of the bulk material is defect- and dislocation-free. Future development of MC-CMP is promising for fabricating wavefront-preserving and ultra-stable channel-cut monochromators, which are crucial to exploit the merits of fourth-generation synchrotron radiation sources or hard X-ray free-electron lasers.

## Abstract 2

The novel magnetically controlled chemical–mechanical polishing technique features simplicity in mechanical components and compatibility with almost all kinds of surface shapes, and is able to fabricate high-accuracy inner-wall surfaces without damaging layers.

## 1. Introduction

Crystal optics, as one kind of key optical component, are extensively employed in hard X-ray beamlines (>3 keV) to obtain monochromatic synchrotron radiation through single-crystal diffraction of X-ray photons. Crystal monochromators are indispensable components of hard X-ray beamlines performing, namely, diffraction, imaging and spectroscopy experiments (Bergeard *et al.*, 2011 ▸; Pankratov & Kotlov, 2020 ▸; Beckhoff *et al.*, 2007 ▸; Okamura *et al.*, 2010 ▸; Chan *et al.*, 2020 ▸). Different techniques place distinctive requirements on the crystal monochromators. Some of the experiments’ demands for high energy resolution, *e.g.* for inelastic X-ray scattering, nuclear resonance scattering, resonance inelastic X-ray scattering, *etc*., require energy-resolving power at different levels (Cunsolo *et al.*, 2012 ▸; Albert *et al.*, 2011 ▸; Paliwal *et al.*, 2021 ▸). Those beamlines with highly focused beams demand high position stability of the beam spot which is highly affected by the crystal monochromators (Juanhuix *et al.*, 2014 ▸; Kunz *et al.*, 2009 ▸; Aragão *et al.*, 2018 ▸). For imaging or coherence purpose beamlines, homogeneity of the crystal monochromators is requested to preserve the wavefront of the beam through propagation (Zozulya *et al.*, 2014 ▸; Schlueter *et al.*, 2019 ▸; Rau *et al.*, 2011 ▸; Cocco *et al.*, 2022 ▸).

Among all crystal optics, the double-crystal monochromator (DCM) is the workhorse at hard X-ray beamlines. However, multiple crystal arrangements suffer from the unavoidable mechanical fluctuation of separate crystals (Diaz *et al.*, 2010 ▸; Sergueev *et al.*, 2016 ▸). Vibrations in the beam position at samples and a decrease in coherent flux will be caused by angular instabilities. Channel-cut crystal monochromators are promising for extremely high structural stability. Channel-cut crystals are fabricated on monolithic blocks with two inner surfaces oriented precisely parallel and demonstrate high angular stability for most applications. One of the critical challenges in fabricating channel-cut crystals is due to the notoriously ‘narrow working space’ – the space between the two inner surfaces of the crystals of the single monolithic block. Usually, fine polishing of channel-cut crystals can only be realized by highly skilful experts working manually with great caution. Consequently, productivity is low and reproducibility is unpredictable.

Fourth-generation synchrotron radiation sources feature a near-diffraction-limit emittance and high flux of coherent photons. The High Energy Photon Source, as an example, is a fourth-generation green-field synchrotron radiation source under construction with ∼60 pm rad emittance and a high fraction of coherent photon flux (Jiao *et al.*, 2018 ▸, 2020 ▸; Tao, 2019 ▸). To make good use of the unprecedented quality of the photons generated from the source, optical components are strictly required to ensure stability (*e.g.* position, angle) as well as to preserve the coherent fraction of X-ray photons. Regarding channel-cut crystals, compactness and high stability are two important merits that could be beneficial for beamlines at fourth-generation synchrotron radiation sources or free-electron lasers. The most critical issue is to preserve the wavefront, meaning that the diffraction surface of the crystal should be damage-free and highly polished.

Various machining technologies have been developed for fabricating channel-cut optics. Firstly, the wet chemical etching (WCE) method has been widely used to remove damaged layers of crystal surfaces by chemical etching. The only drawback of WCE is that it introduces large fluctuations of roughness and flatness on the crystal surface (Steinert *et al.*, 2006 ▸). Secondly, the traditional chemical–mechanical polishing (CMP) method has been successfully employed to process the inner walls of channel-cut crystals with highly improved surface accuracy (Khachatryan *et al.*, 2004 ▸; Kasman *et al.*, 2015 ▸, 2017 ▸). Nevertheless, owing to unavoidable scratches and defects induced by traditional CMP, a sufficiently flat and smooth surface is extremely difficult to generate within a reasonable amount of time. Lastly, state-of-the-art plasma chemical vaporization machining (PCVM) (Hirano *et al.*, 2016 ▸; Katayama *et al.*, 2019 ▸), a purely ion chemical method, has been developed to produce near-perfect polished surfaces with high accuracy and no damage. However, PCVM has been mostly applied for fabricating high-quality mirrors with strict technical requirements and strikingly high cost. Therefore, PCVM is not a cost-effective approach for making channel-cut crystal optics.

In this work, we report a novel and economically affordable approach for fabricating silicon-based channel-cut crystal optics. The so-called magnetically controlled chemical–mechanical polishing (MC-CMP) method is developed specifically for polishing silicon-based channel-cut optics with a gap of a few millimetres. The MC-CMP approach is a cost-effective and computer-controlled approach. With this approach, we have fabricated a batch of channel-cut crystals. A few of the channel-cut crystal optics were polished to be used for highly stable wavefront-preserving monochromators. The performances of the channel-cut crystals were characterized using either an off-line white-light profiler, high-resolution transmission electron microscopy or on-line diffraction and topography.

## 2. Description of the approach

### 2.1. The MC-CMP system

**Figure 1:**
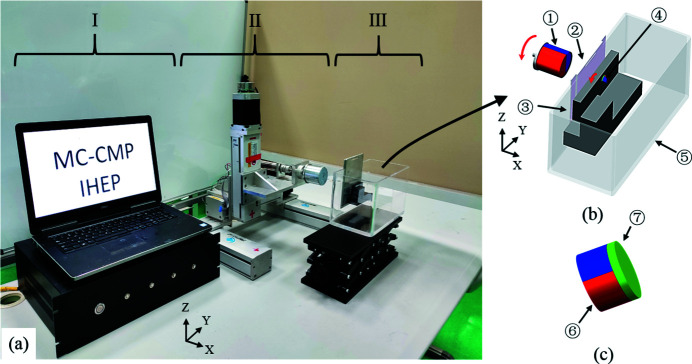
(*a*) Photograph of the MC-CMP system, showing (I) the computer and driver, (II) the gantry guide rail and rotating mechanism of the driving magnet, and (III) the vessel system. (*b*) Details of the vessel system. (*c*) Small polishing magnet. Major components are numbered: (1) large magnet and its motion mechanics including rotation and *X/Y/Z* rectilinear motion; (2) pad which can be inserted into the vessel; (3) channel-cut crystal that can be mounted on the pad (2); (4) small polishing tool; (5) vessel filled with slurry or polishing solution; (6) mini magnet; (7) covering layer; additional material can be added on the mini magnet when different processes are carried out.

**Figure 2:**
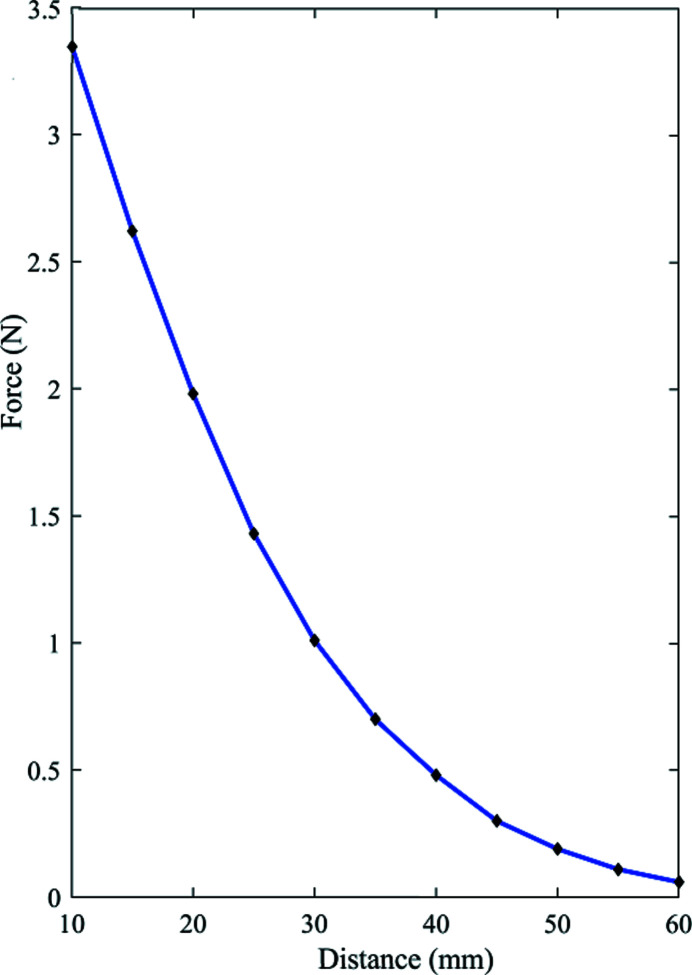
Comparison of the gap-dependence of the magnetic force. Experimental values and a fitting curve are shown by black dots and the blue solid line, respectively.

The MC-CMP system, as shown in Fig. 1 ▸, consists of three major components: (*a*) a mini cylinder magnet in a vessel filled with slurry or polishing solution; (*b*) a gantry guide rail and rotating mechanics for the driving magnet; and (*c*) a control computer. Rotation of the large cylinder magnet is achieved by a DC motor while the three-dimensional translation movements *X*/*Y*/*Z* are implemented through a gantry guide rail. Both magnets are radially magnetized; the rotation and *Y*/*Z* rectilinear motion of the small polishing tool is driven by an alternating magnetic field applied by the large moving external magnet. The stress can be automatically controlled by varying the distance along the *X*-axis – Fig. 2 ▸ shows the relationship between the distances of the magnets and the magnetic force in the static condition – whereas it becomes much more complex when the magnets are rotating. Detailed illustrations are discussed in Section S1 of the supporting information. Eventually, owing to the small polishing magnets, it is highly feasible to fabricate channel-cut crystals with small gap (shown in the next section).

### 2.2. Fabrication of channel-cut (CC) crystals

Two Si(111) CC crystals with 7 mm gap were fabricated from a floating-zone silicon single-crystal ingot. The optical face was cut with an orientation accuracy of 0.05°(*X*) and 0.1° (*Y*). A detailed drawing and the sizes of the channel-cut crystals are given in Fig. S5 and Table S3 of the supporting information.

After grinding using a 80 µm vitrified bond diamond/CBN wheel, one of the two CC crystals was directly immersed in HNO_3_/HF (10:1) mixtures over 40 min to completely remove damaged layers. Eventually in total a 100 µm-thick damaged silicon layer was removed after etching. The other CC crystal was fabricated using the MC-CMP approach in four steps:

(1) Rough mechanical polishing. A small polishing tool covered with cast iron material was applied; the slurry, consisting of alumina solution with particle size 10 µm, was stirred continuously during processing. Taking the first reflection inner wall as an example, the processing time took about 16.2 h and a nearly 120 µm-thick layer was removed. The optimized rotation speed and the distance between the magnets were 200 r.p.m. and 25 mm, respectively.

(2) Rough CMP polishing. Polyurethane (Sciyea EP002) with 90-shore hardness was chosen as the covering layer instead of cast iron. The slurry was replaced by an alumina suspension with particle size of 3 µm (pH = 10). This process took about 32.3 h and a thickness of ∼55 µm was removed. For this process, the optimized rotation speed and distance between magnets were 250 r.p.m. and 30 mm, respectively.

(3) Wet chemical etching. The CC crystal from step 2 was etched in HNO_3_/HF (10:1) for 12 min. In this step, in total a 35 µm-thick layer was removed.

(4) Final CMP polishing. The covering layer on the small tool was changed to polyurethane (Sciyea FSP13) with 66-shore hardness; the CC crystal from step 3 was submerged in silica suspension (pH = 11) with particle size of 50 nm for polishing. In this step, a thickness of nearly 8 µm was removed during 97 h of polishing; the corresponding rotation speed and distance between magnets were 350 r.p.m. and 40 mm, respectively.

A photograph of the final channel-cut crystal after MC-CMP processing is shown in Fig. S8; further technical information about the processing is shown in Section S2 of the supporting information

## 3. Experimental methods for characterization

To characterize the details of the surface and micro-structural changes of the material, we carried out two optical investigations using a white-light profiler (WLP; type ZYGO SER 4405A) and high-resolution transmission electron microscopy (HRTEM; type FEI Talos F200x), respectively. Details of the fabrication of the HRTEM test specimen can be found in the supporting information.

**Figure 3:**
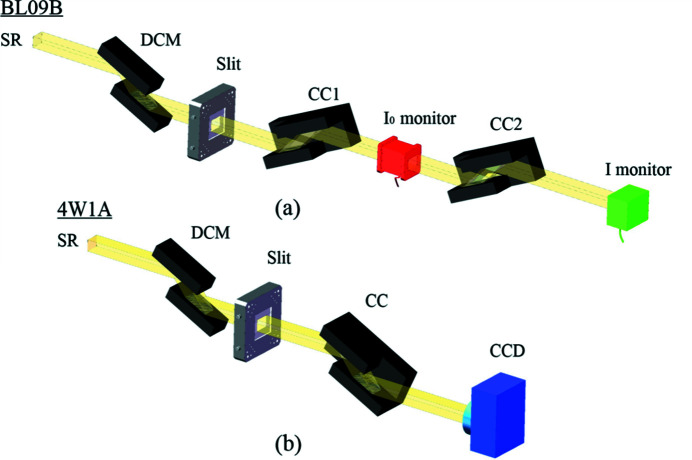
Schematic view of the experimental setup for (*a*) rocking-curve and reflectivity measurements at BL09B (SSRF) and 1B3B (BSRF) (opening size of slit: 5 mm horizontal, 1 mm vertical) and (*b*) X-ray topography measurements at 4W1A (BSRF) (opening size of slit: 5 mm horizontal, 5 mm vertical).

For crystal optics, it is important to conduct X-ray topography or rocking-curve measurements. In Fig. 3 ▸, the optical layouts for on-line X-ray characterization measurements are shown. The rocking curve and reflectivity were measured at beamlines BL09B, Shanghai Synchrotron Radiation Facility (SSRF), and 1B3B, Beijing Synchrotron Radiation Facility (BSRF). In this experiment [Fig. 3 ▸(*a*)], the (+, −, −, +, −, +) crystal configurations were adopted with double flat crystals and a pair of CC crystals. An X-ray topography experiment was conducted at beamline 4W1A (BSRF). In this experiment [Fig. 3 ▸(*b*)], the (+, −, +, −) configuration was adopted. Both beamlines were equipped with Si(111) double-crystal monochromators. The intensity of the beam was measured using an ionic chamber or photodiode. For imaging, a HAMAMATSU (Model C13440-20CU) CCD was used. The CCD consisted of 2048 × 2048 pixels (6.5 µm per pixel) and 4× objective lens.

## 4. Results and discussions

**Figure 4:**
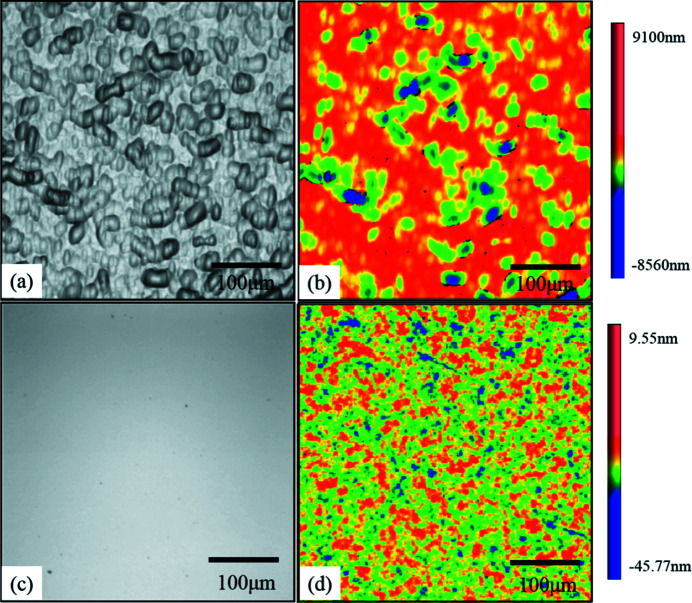
(*a*) Optical micrograph of the WCE CC. (*b*) The corresponding roughness is 873.9 nm r.m.s. in a 417.19 µm × 417.19 µm area. (*c*) Optical micrograph of the MC-CMP CC. (*d*) The roughness is 0.614 nm RMS in a 417.19 µm × 417.19 µm area.

For the CC crystal from WCE, numerous and large-size speckles were found on the surface [Fig. 4 ▸(*a*)], and the roughness value of the etched surface was 873.9 nm r.m.s. [Fig. 4 ▸(*b*)]. By contrast, for the MC-CMP CC crystal, the obtained surface was homogeneous without any observable speckle and scratches [Fig. 4 ▸(*c*)], and a substantially high surface roughness of 0.614 nm r.m.s. was measured [Fig. 4 ▸(*d*)].

**Figure 5:**
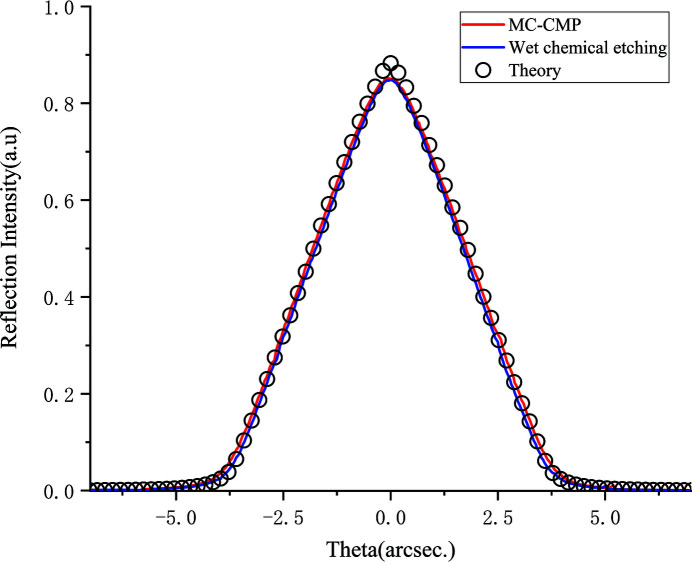
Comparison of the experimental and theoretical rocking curves for MC-CMP and WCE processed channel-cut crystals. The experimental data are shown as solid lines (red and blue lines correspond to MC-CMP and WCE processed channel-cut crystals, respectively). The black hollow circles represent theoretical calculation.

The rocking curve and reflectivity are highly influenced by the perfectness of the lattice of the crystal diffraction volume. As shown in Fig. 5 ▸, rocking curves at 15 keV were measured for both WCE CC and MC-CMP CC crystals and compared. The Darwin width for both WCE and MC-CMP CC crystals agreed well with the theoretical calculations based on dynamical diffraction theory. Meanwhile, the peak reflectivity of the MC-CMP CC and WCE CC were 85.1% and 84.8%, respectively, which are close to the theoretical value of 88.3%. The experimental rocking curves are very close to theoretical values, indicating that the diffraction volume is a perfect crystal and damaged layers are removed by both the MC-CMP or WCE processes. On the other hand, the diffraction surface is well preserved when using the MC-CMP method for polishing the inner faces of CC crystals.

**Figure 6:**
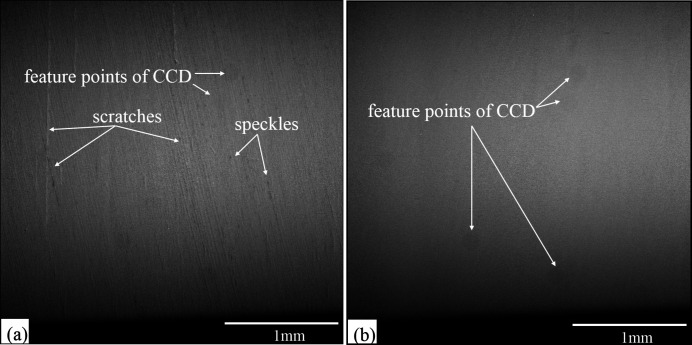
(*a*) Topography of the etching surface. Numerous scratches and speckles were introduced on the surface. (*b*) Topography of the polishing surface. The spot is balanced – a few dark points originate from the CCD.

Fig. 6 ▸ shows X-ray topography images of the crystal surface for the WCE CC and MC-CMP CC crystals. There are several scratches on the WCE CC crystals, but the MC-CMP CC crystal is quite homogeneous and scratch-free.

**Figure 7:**
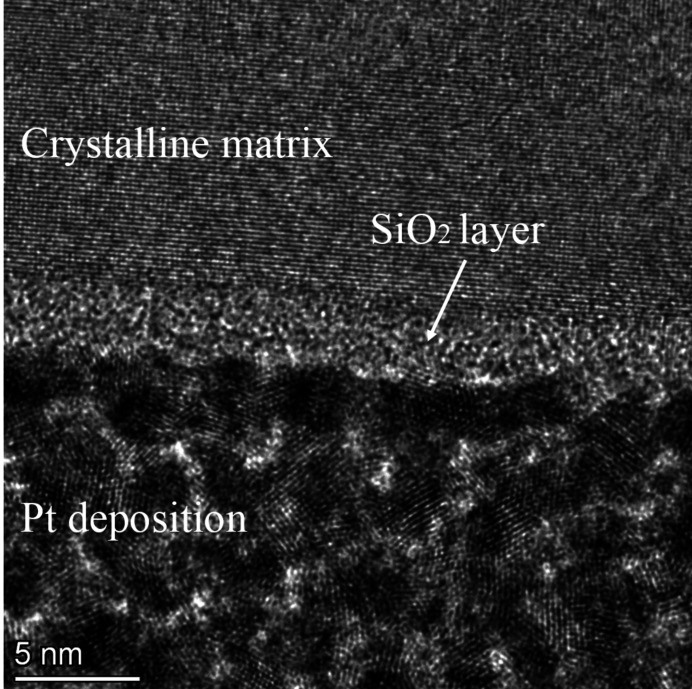
Cross-section HRTEM image of the polishing CC workpiece. A three-tiered structure is displayed: the bottom is made of Pt deposition, the middle is an SiO_2_ layer, and the top is crystalline matrix.

Detailed elemental mapping using energy-dispersive X-ray spectroscopy (EDX) can be found in Fig. S13. According to the EDX mapping, a SiO_2_ layer with a uniform thickness of about 2.5 nm was found on the surface of the perfect crystalline matrix. After final processing treatment with small grain size and processing pressure, there is no amorphous layer. By contrast, in the traditional CMP method, heat and stress are highly concentrated and some surface silicon turns into an amorphous form (Xu *et al.*, 2007 ▸; Zhang & Zarudi, 2001 ▸). By contrast, the MC-CMP approach applies a tiny amount of stress and negligible local heat and therefore suppresses the crystal-to-amorphous transformation, though the amorphous form would appear if using large grain size and high processing forces. Using the MC-CMP method, the SiO_2_ layer (Fig. 7 ▸) with nanometre-scale thickness and uniform construction obtained on a perfect crystalline matrix could be beneficial for preserving the wavefront and coherence of high-quality photons from fourth-generation synchrotron radiation sources or free-electron lasers.

## 5. Conclusion and perspective

We have developed the magnetically controlled chemical–mechanical polishing (MC-CMP) approach for fine polishing silicon-based channel-cut crystals with small gaps. The polishing can be precisely controlled by a small polishing tool by applying a magnetic field. The MC-CMP features simplicity in mechanical components and compatibility with almost all kinds of surface shapes with variable polishing tools; hence it can be applied to polish channel-cut crystal optics with a thin gap narrower than the workable space for a human hand. Both offline (WLP and HRTEM) and online synchrotron measurements showed a high-accuracy roughness of the inner-wall surfaces without damaging layers. Further, similar to the small polishing head technology in optical manufacturing, MC-CMP inherently possesses a stable removal functionality, and thus can be used for correcting surface flatness errors of the inner surfaces of the crystals. Eventually, this novel technique could be readily applied at almost every crystal optics laboratory to manufacture channel-cut monochromators for any hard X-ray beamline at either fourth-generation synchrotron radiation sources or free-electron lasers.

## 6. Related literature

The following references, not cited in the main body of the paper, have been cited in the supporting information: Akoun & Yonnet (1984 ▸); Buschow & Boer (2013 ▸); Mahoney & Abbott (2014 ▸, 2016 ▸); Mahoney *et al.* (2013 ▸).

## Supplementary Material

Further details on force analysis, processing, area removal and HRTEM. Tables S1 to S5. Figures S1 to S13. DOI: 10.1107/S1600577522011122/yi5130sup1.pdf

Original test data and theoretical calculation data. DOI: 10.1107/S1600577522011122/yi5130sup2.xlsx
