# Supplementary material for: A magnetically controlled chemical–mechanical polishing (MC-CMP) approach for fabricating channel-cut silicon crystal optics for the High Energy Photon Source
Source: J Synchrotron Radiat. 2023 Jan 1;30(Pt 1):84–9. doi: 10.1107/S1600577522011122 (PMC9814062; doi:10.1107/S1600577522011122)
Supplement: Supplementary file 1 [file s-30-00084-sup1.pdf]

JOURNAL OF SYNCHROTRON RADIATION

Volume 30 (2023)

Supporting information for article:

A magnetically controlled chemical-mechanical polishing (MC-CMP) approach for fabricating channel-cut silicon crystal optics for the High Energy Photon Source

Zhen Hong, Qianshun Diao, Wei Xu, Qingxi Yuan, Junliang Yang, Zhongliang Li, Yongcheng Jiang, Changrui Zhang, Dongni Zhang, Fang Liu, Xiaowei Zhang, Peng Liu, Ye Tao, Weifan Sheng, Ming Li and Yidong Zhao

S1. Force analysis

The pure theoretical calculation of the relationship between the distance of magnets or the processing pressure is complex and requires substantial computation [1,2]. For the purpose of this work, we performed both experimental measurement and theoretical simulations.

The distance dependent magnetic forces were measured using the experimental setup, shown in Fig.S1. The small magnet was amounted on the trigger rod of force sensor (freud DY920, immune to magnetic field)which was placed on the aluminum bracket. Two cylindrical magnets were coaxially arranged. Note that both the cover and trigger rod are made up of aluminum alloy and 316 non-magnetic stainless steel, respectively. The measured results are shown in Tab.S1

Theoretical simulation were conducted to simulate the relationship between the forces and distances of magnets using Comsol Multiphyics 5.4. The NbFeB-N52 magnet was used and the setting parameters are shown in Tab. S2

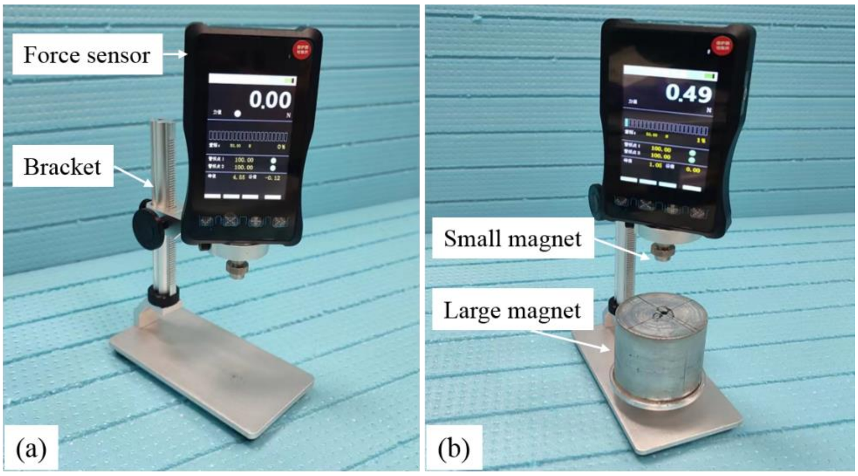

Figure S1 Picture of force measurement setup (a) Setting the force sensor to zero after small magnet amounted on; (b) Put the large magnet under the small magnet.

Table S1 Distances between magnets and measured magnetic forces.

Distance(mm)	10	15	20	25	30	35	40	45	50	55
Force(N)	3.35	2.62	1.98	1.43	1.01	0.7	0.48	0.3	0.19	0.11

Table S2 Setting parameters for NbFeB-N52 magnet using Comsol Multiphyics 5.4.

Options	Model	Relative permeability (air)	Relative permeability (magnet)	Remanent Flux density (big magnet)	Remanent Flux density (small magnet)
Values	mfnc	1.0	1.05	(X,Y,Z)=(0,0,1.45)T	(X,Y,Z)=(0,0,-1.45)T

In Fig. S2, we compared the measured value and the theoretical simulations. For the magnetic forces as a function of the distance between two magnets. Overall, the magnetic force decreases with the increasing distances between the two magnets in both experimental measurements and theoretical simulations. The simulated force values are slightly higher than the measured value. This might be due to the simplified model used in the simulation or the uncertainty of the measured value. Qualitatively, the maximum magnetic force is about 3.4 N when the distance between two magnets reaches 10mm; while the magnetic force decreased down to 0.5N when the distance is about 40mm. It should be note that the force-distance relationship is correct only if the two magnets are relatively stationary.

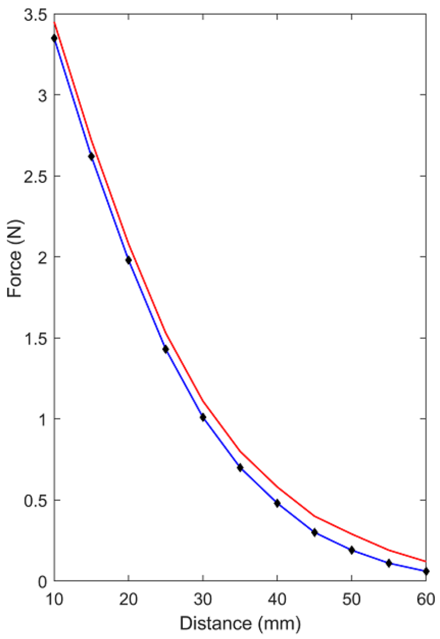

Figure S2 The relationship between the forces and distances of magnets the black points represent the values in Tab .S1, the blue solid line is the fitting curve of those points, the red solid is derived from Comsol Multiphyics 5.4.

In practice, when the magnets are rotating, there exist a hysteresis angle α due to the friction between the covering layers and the work-piece as depicted in Fig.S3 (b).

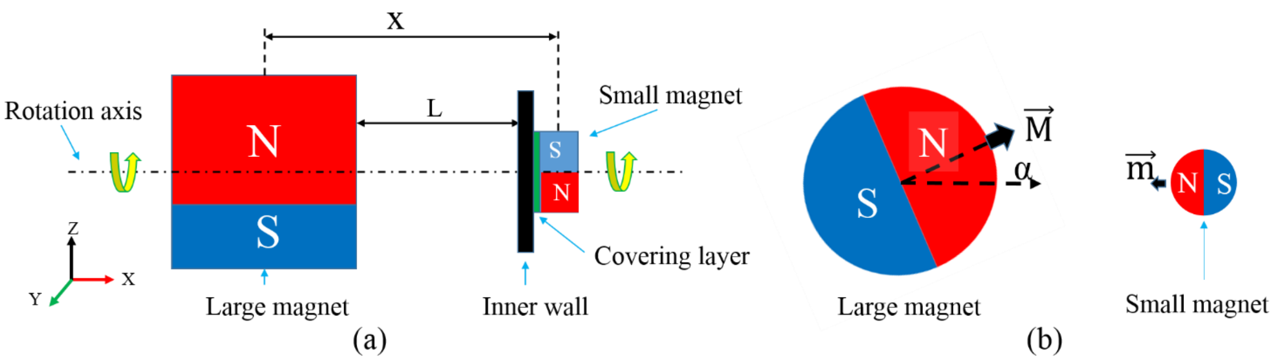

Figure S3 (a) Y axis direction view of magnets when rotating; (b) X axis direction view.

The smooth operation of the magnet needs to meet the force balance condition

$$\begin{cases} \overrightarrow{F_n} + \overrightarrow{F_m} = 0 \\ \overrightarrow{T_f} + \overrightarrow{T_m} = 0 \end{cases}$$

$\overrightarrow{T_f}$ is the resistance moment, $\overrightarrow{T_m}$ is the magnetic driving moment, $\overrightarrow{F_n}$ is the supporting force, F_m the magnetic force, as shown in Fig.S4

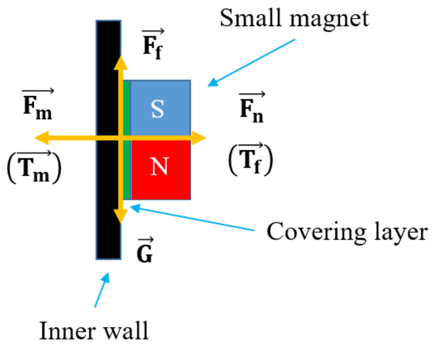

Figure S4 The illustration of force between the inner wall and covering layer.

When the distance between the two magnets is far enough, the magnetic dipole displacement method model can be used for calculation[3-5],for easy to explanation, the method mentioned above is used, so

$$\overrightarrow{F_m}=\frac{3\mu_0|\mathrm{M}||\mathrm{m}|\cos\alpha}{4\pi x^4} \quad \ldots\ldots\ldots\ldots\ldots\ldots\ldots\ldots\ldots\ldots\mathrm{I}$$

$$\overrightarrow{T_m}=\vec{m}\times\vec{B}= \frac{\mu_0|\mathrm{M}||\mathrm{m}|\sin\alpha}{4\pi x^3}\ldots\ldots\ldots\ldots\ldots\ldots\ldots\mathrm{II}$$

$$\overrightarrow{T_f}=\frac{2\lambda\times(\overrightarrow{F_n}\times r)}{3}\ldots\ldots\ldots\ldots\ldots\ldots\ldots\ldots\ldots\mathrm{III}$$

μ_0 is a constant named vacuum permeability，|M| is magnetic dipole moment of large magnets，|m| is magnetic dipole moment of small magnets, when the magnet type and shape are determined, |M| and |m| are also constant, x is the distance from the center of geometric symmetry of the large magnet to the center of geometric symmetry of the small magnet, λ is the friction coefficient between covering layer and workpiece.

Combine the above three formulas (I-III), α can be calculated by the equation below:

$$\alpha=\tan^{-1}\frac{2\lambda r}{x}$$

λ will be different when the surface roughness varies, so the values of α and magnet forces F_m are dynamic during processing even though the distance between magnets is fixed.

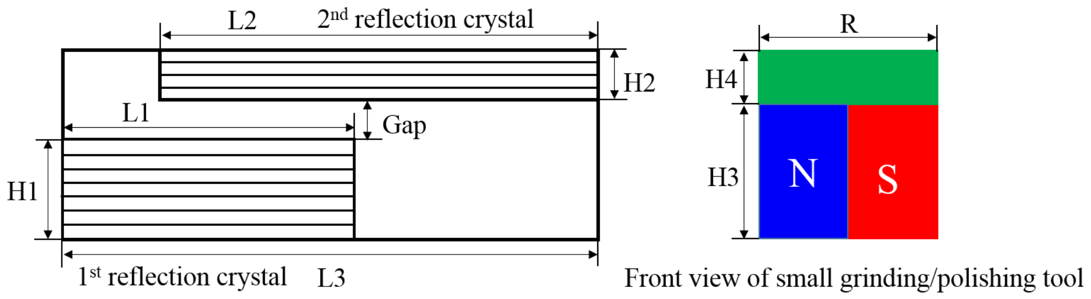

Figure S5 (left) Top view of the channel-cut crystal; (right) Front view of magnetic polishing tool; the size as indicated are listed in Table S4. Note that the external driving force is applied by a 60 mm-diameter and 30 mm-high cylinder-like magnet.

Table S3 Dimensions as specified in Fig. S5.

Dimension	L1	L2	L3	H1	H2	Gap	H3	H4	R
Size(mm)	60	90	110	20	10	7	4	2	8

*The width of reflection surface is 30mm.

**Effective polishing area: 1st reflection crystal @55 by 25 mm²; 2nd reflection crystal @85 by 25 mm².

S2. Processing Details

It is well known that there often exist deep damage layer with high roughness in silicon after grinding. As shown in Fig.S6, a cast iron as covering layer on top of the magnets, is applied to remove the damage layer during rough mechanical polishing. Moreover, certain grooves were cut on the surface of the cast iron for facilitating the

slurry to access the interface between covering layer and work-piece. As surface roughness is improved, friction between cast iron and silicon decreases gradually thus the small polishing tool becomes unstable when rotating. It is then time to perform the fine mechanical polishing using the polyurethane with high-shore hardness as the covering layer.

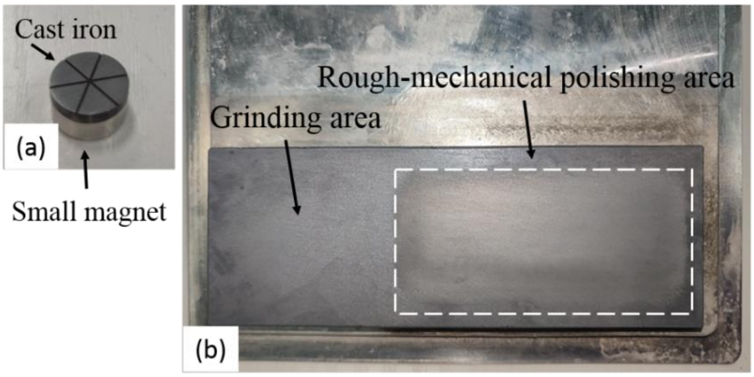

Figure S6 (a) Small polishing tool with cast iron covering layer; (b) Rough-mechanical polishing area.

All the processing routes of small polishing tool are similar to raster scan as shown in Fig.S7, while the distance of step-by-step, rotation speed and the distance of magnets can be adjusted differently in every procedure.

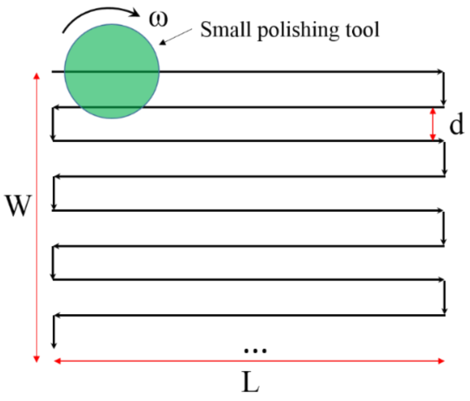

Figure S7 Scanning processing routes, where L, W and d refers to length, width and distance, respectively and ω is the rotation speed of small polishing tool.

The effective polishing area is defined as the area of processing routes given in Table S3. The minimum processing time can be estimated by

$$T(\mathrm{min}) = \frac{W + L \cdot \left(\frac{W}{d} + 1\right)}{V_y} \cdot N$$

Where V_y represents the speed along Y axis, N is the number of circulation, W, L and d are shown in Fig.S7. A set of parameters are shown in Table S4.

Table S4 Processing parameters.

Step	d(mm)	W(mm)	L(mm)	V_y(mm/min)	X(mm)-distance of magnets	N
1)	1	55	25	15	25	10
2)				15	30	20
3)				15	40	60

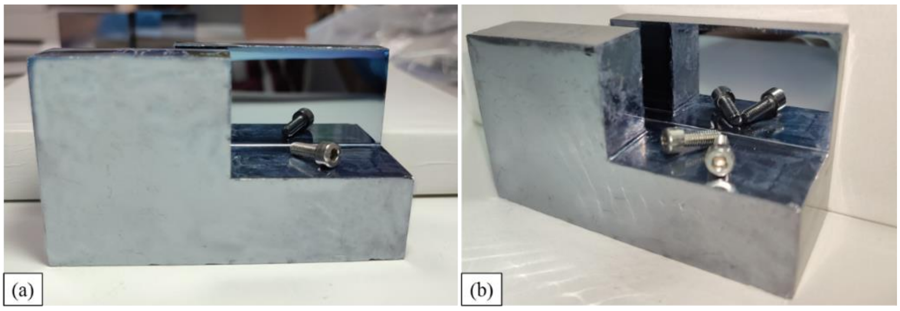

Figure S8 Photo of channel-cut crystal after MC-CMP polishing (a) front view; (b) side view; the screws are mirrored clearly by the polished mirror-like surface of the channel-cut crystal.

S3. Overlapping and non-overlapping areas removal

As shown in Fig.S9, we designed a mechanical structure made of aluminum alloy to mimic the grooved crystal, so as to perform optical metrology (ZYGO Verifire QPZ) during processing. In this mechanical structure, the two vertical walls can be easily assembled and disassembled with aluminum alloy screws. Two 1mm-thick silicon wafers with (111) orientation were glued on the aluminum bases, the gap between the surfaces of wafers is 7mm. The SiO_2 suspension (pH=11) with 50 nm particle was used as polishing solution.

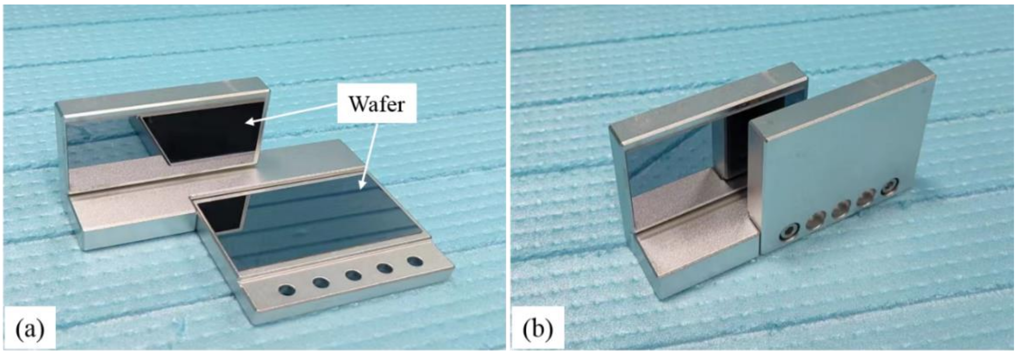

Figure S9 (a) photo of 'channel-cut crystal' made of aluminum; (b) 'channel-cut' after assembly.

The relationship between volume removal rates and processing parameters (i.e. rotation speed, distance between magnets) is experimentally determined. Firstly, we changed the rotation speed for fixed distance of 40mm. Secondly, we change the distance for fixed rotation speed at 300 rpm. The parameters and measured results are summarized in Table.S5. Generally, the volume removal rate (VRR) increases as almost linearly with the increasing rotation speed. By contrast, the VRR decreases with the increasing distance.

In Figure.S10, the removal footprints are compared. Firstly, the thickness of the removed layer increases with the increasing rotation speed. Secondly, owing to the acceleration, deceleration and uneven polishing areas in the beginning or near the end, the removed thickness is smaller in these parts in compare with the middle area when the speed is constant. Thirdly, the removal rates in overlapping and non-overlapping

areas are almost the same, suggesting that the difference of slurry flow rates has little impact on removal rate.

Table S5 Experimental parameters and result for volume removal rate.

Volume removal rate vs. rotationspeed					
Rotation speed (rpm)	200	250	300	350	400
volume removal rate (mm^3/min10^{-3})	0.922	1.279	1.493	1.805	1.966
Other parameters	V_y=15mm/min, $L_{routine}$=20mm, Distance=40mm				
**Volume removal rate vs. distance**					
Distance (mm)	30	35	40	45	
volume removal rate (mm^3/min*10^{-3})	3.005	1.98	1.529	1.123	
Other parameters	V_y=15mm/min, $L_{routine}$=50mm, Rotation speed=300rpm				

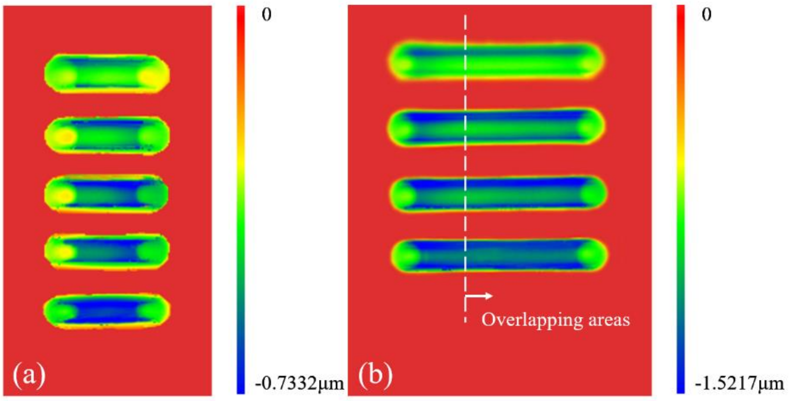

Figure S10 Removal footprints of polished silicon surface. (a) with respect to rotation speed; from top to bottom, rotation speeds are 200 rpm,250rpm,300rpm,350rpm,400rpm respectively; (b) with respect to distance,from top to bottom, distances are 30mm,35mm,40mm,45mm separately.

We plotted the experimental data in Fig.S11 to show the relationship between volume removal rate and rotation speed, distances of magnets separately, it can be found that there is nearly a linear relationship between the rotation speed and volume removal rate (V_y is small, it has little effect on relative velocity) , so the classical Preston equation can be (approximately) applied for this experiment.

$$\Delta Z=\int_0^t K_p vpdt$$

Where, ΔZ is the volume removal in t period, t is 1min in the measurements, V represents the relative speed of small tool, p is the pressure between covering layer and inner wall of channel cut.

By contrast, the relationship between the volume removal rates and the distances cannot be fitted using function based on the relative curve between distances and forces under stationary situation (We assumed that the volume removal rate and pressure are also linear relationship). The major reason is due to the varying hysteresis angle α as the distance of magnets change. More detailed analysis of the magnetic force is out of the scope of this paper and will be carried out in future works.

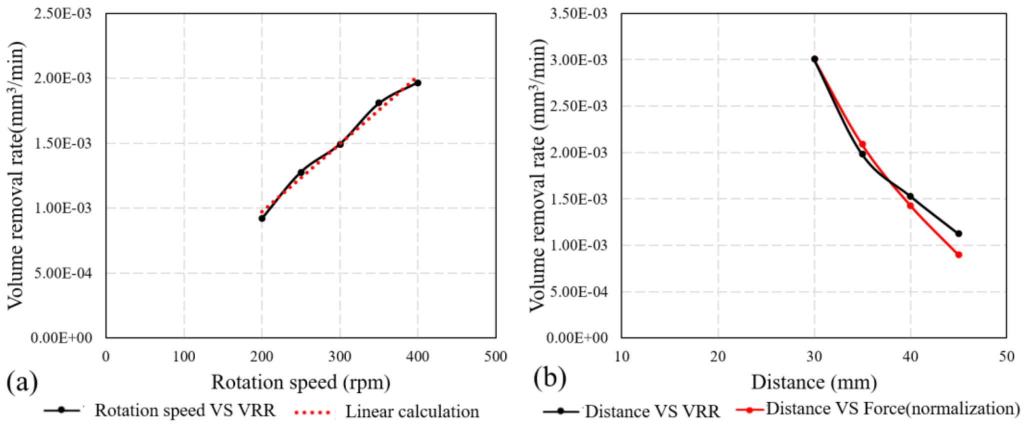

Figure S11 Plots for volume removal rate as a function of (a) rotation speed at fixed distance; (b) distance at fixed rotation speed.

S4. HRTEM

To examine the microstructure of the crystal, a sample piece ($5(L) \times 3(W) \times 2(T)$ mm^3) was cut from the fabricated channel-cut crystals using diamond wire. After depositing Pt on the surface of the sample, the FIB (Focused Ion Beam) technology was used to prepare the specimen ($15(L) \times 12(W) \times 0.03(T)$ μm^3) suitable for High Resolution Transmission Electron microscopy (HRTEM).

Fig. S12 shows the specimen for HRTEM at different scales under microscope. In order to protect the surface of the sample from damage by the ion beam bombardment, two layers of Pt were deposited on the surface of crystal. The first layer (Fig. S12(d) Pt layer 1) in thin enough with slow deposition speed and the second layer ((Fig. S12(d) Pt layer 2))is quickly deposited to achieve sufficient thickness.

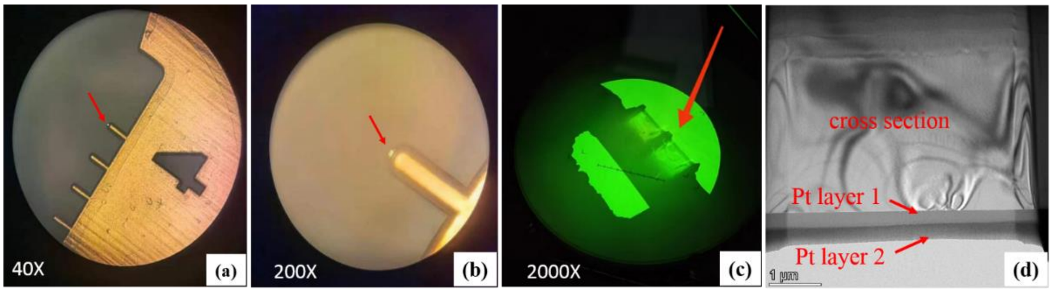

Figure S12 Microscope view of the HRTEM specimen at different magnifying ratios. The scale bars are shown in the inset.

Fig. S13 shows the high resolution morphology of the specimen and elemental mapping by Energy Dispersive X-Ray spectroscopy (EDX). On the top surface of the bulk silicon crystal, there is a thin layer (~2.5nm thick) of SiO_2 (Fig. S13(b)). The Pt layer locates on top of the SiO_2 layer (Fig. S13(d)).

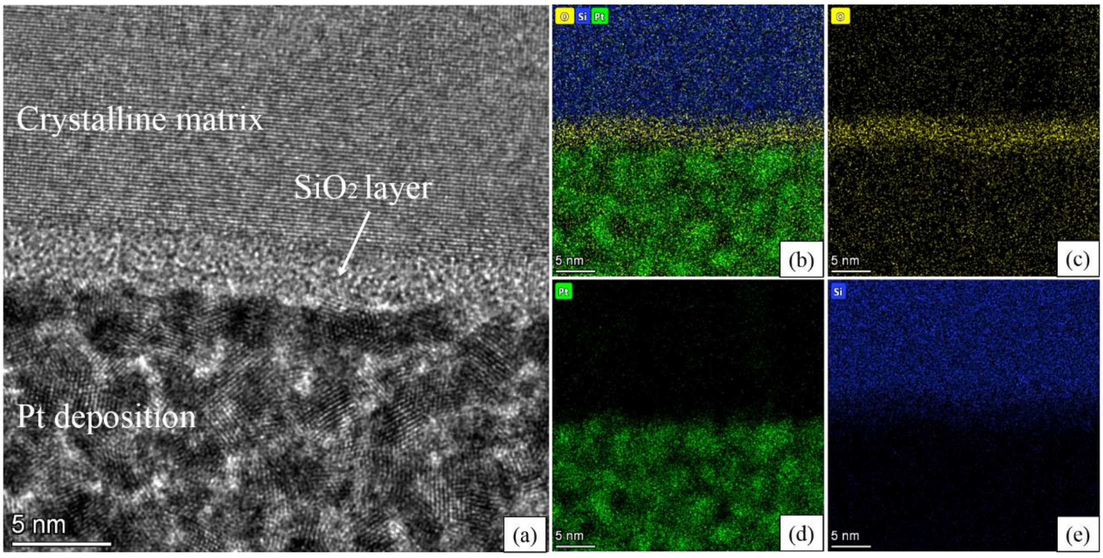

Figure S13(a) HRTEM micrograph of the cross section of the FIB fabricated specimen; from top to bottom are the silicon matrix, a thin layer of SiO_2 and the deposited protecting Pt layer, respectively. (b) Energy Dispersive X-Ray spectroscopy (EDX) map of the sample showing the existence of three elements; (c) the EDX map of O; (d) the EDX map of Pt; (e) the EDX map of Si.

References

[S1]. Akoun G, Yonnet J P. 3D analytical calculation of the forces exerted between two cuboidal magnets[J]. IEEE Transactions on magnetics, 1984, 20(5): 1962-1964.

[S2]. Buschow K H J , Boer F R D . Physics of Magnetism and Magnetic Materials[M]. World Book Publishing Company Beijing Branch, 2013.

[S3]. Mahoney A W, Wright S E, Abbott J J. Managing the attractive magnetic force between an untethered magnetically actuated tool and a rotationpermanent magnet[C]//2013 IEEE International Conference on Robotics and Automation. IEEE, 2013: 5366-5371.t

[S4]. Mahoney A W, Abbott J J. 5-DOF Manipulation of an Untethered Magnetic Device in Fluid using a Single Permanent Magnet[C]//Robotics: Science and Systems. 2014.

[S5]. Mahoney A W, Abbott J J. Five-degree-of-freedom manipulation of an untethered magnetic device in fluid using a single permanent magnet with application in stomach capsule endoscopy[J]. The International Journal of Robotics Research, 2016, 35(1-3): 129-147.
